# Pluripotent stem cell-derived skeletal muscle fibers preferentially express myosin heavy-chain isoforms associated with slow and oxidative muscles

**DOI:** 10.1186/s13395-020-00234-5

**Published:** 2020-06-03

**Authors:** Tania Incitti, Alessandro Magli, Asher Jenkins, Karena Lin, Ami Yamamoto, Rita C. R. Perlingeiro

**Affiliations:** 1 Lillehei Heart Institute, Department of Medicine, University of Minnesota, 4-128 CCRB, 2231 6th St. SE, Minneapolis, MN 55455 USA; 2 Stem Cell Institute, University of Minnesota, Minneapolis, MN USA

**Keywords:** Pluripotent stem cells (PSC), Skeletal myogenesis, Myofibers, Fiber types, Fast, Slow, Pax3, Pax7, Engraftment, Muscle stem cell

## Abstract

**Background:**

Skeletal muscle function is essential for health, and it depends on the proper activity of myofibers and their innervating motor neurons. Each adult muscle is composed of different types of myofibers with distinct contractile and metabolic characteristics. The proper balance of myofiber types is disrupted in most muscle degenerative disorders, representing another factor compromising muscle function. One promising therapeutic approach for the treatment of these diseases is cell replacement based on the targeted differentiation of pluripotent stem cells (PSCs) towards the myogenic lineage. We have previously shown that transient induction of Pax3 or Pax7 in PSCs allows for the generation of skeletal myogenic progenitors endowed with myogenic regenerative potential, but whether they contribute to different fiber types remains unknown.

**Results:**

Here, we investigate the fiber type composition of mouse PSC-derived myofibers upon their transplantation into dystrophic and non-dystrophic mice. Our data reveal that PSC-derived myofibers express slow and oxidative myosin heavy-chain isoforms, along with developmental myosins, regardless of the recipient background. Furthermore, transplantation of the mononuclear cell fraction re-isolated from primary grafts into secondary recipients results in myofibers that maintain preferential expression of slow and oxidative myosin heavy-chain isoforms but no longer express developmental myosins, thus indicating postnatal composition.

**Conclusions:**

Considering oxidative fibers are commonly spared in the context of dystrophic pathogenesis, this feature of PSC-derived myofibers could be advantageous for therapeutic applications.

## Background

Adult skeletal muscles are characterized by several major components: myofibers, their innervating motor neurons and muscle resident stem cells, along with fibroblasts, fibro-adipogenic progenitors, endothelial cells, and immune cells [1]. Upon injury or disease, muscle stem cells are activated to regenerate new muscle tissue [2]. Each adult muscle is a heterogeneous combination of different types of myofibers characterized by specific contractile and metabolic properties, which are classified into three main categories: (i) oxidative slow-twitch, expressing type I myosin heavy chain (MyHC); (ii) oxidative fast-twitch, expressing type IIA MyHC; and (iii) glycolytic fast-twitch, expressing type IIX and IIB MyHC isoforms [3]. In mammals, prenatal fibers initially express the embryonic MyHC isoform, followed by fetal/neonatal, type I, and, later on, type II, which is further classified into IIA, IIX, and/or IIB, with the latter not expressed in humans [4]. Developmental MyHC isoforms disappear during the first weeks of postnatal life but the embryonic type becomes re-expressed for a short time window during regeneration in adults, a process that partially recapitulates developmental myogenesis [3]. Even though each muscle acquires a unique pattern of MyHC expression and consequently, fiber type characteristics, their composition is highly plastic and can be influenced by several factors, such as muscle loading, exercise, hormone-mediated signaling, and disease [3]. Moreover, myofibers undergo fiber type transitioning, which generate hybrid myofibers expressing two or more different MyHC isoforms at the same time [5].

Many muscle degenerative disorders, including Duchenne muscular dystrophy (DMD), facioscapulohumeral muscular dystrophy (FSHD), myotonic dystrophies 1 and 2 (DM1-2), and Pompe disease, exhibit disrupted regulation of fiber type composition, thus further compromising muscle function [6]. For instance, in DMD, the fast fibers are preferentially affected while the slow fibers degenerate more slowly, suggesting that dystrophin may have a major role in fibers with the ability to respond to the highest frequency stimulation [7]. One promising therapeutic approach for muscle wasting diseases is cell replacement based on the myogenic differentiation of pluripotent stem cells (PSCs). We developed a doxycycline-inducible system that enables the generation of large numbers of proliferating early skeletal myogenic progenitors from PSCs through the transient expression of the myogenic transcription factors Pax3 or Pax7. We have documented that upon transplantation, these myogenic progenitors give rise to new myofibers, seed the muscle stem cell compartment, provide functional muscle improvement, and remodel their molecular signature to acquire a more mature phenotype [8–10].

A few studies have investigated the fiber type composition of PSC-derived skeletal muscle in vitro [11, 12], but to date, only one report has documented in vivo fiber type characteristics of engrafted tissue, which was generated from PSC-derived teratomas [13]. Since fiber type composition plays an important role in skeletal muscle adaptation to pathological stimuli [7, 14, 15], determining how in vitro-generated PSC-derived myogenic progenitors participate in this process will provide important information for the development of effective cell-based skeletal muscle replacement therapies.

Here, we sought to investigate the fiber type composition of engrafted muscles following the transplantation of PSC-derived myogenic progenitors in a mouse model of DMD. These in vivo studies revealed that PSC-derived skeletal myofibers display a higher proportion of oxidative slow myofibers compared to freshly isolated primary muscle stem cells. These results suggest that PSC-derived myofibers could be beneficial in the context of muscular dystrophies, not only for their regenerative capacity, but also for their ability to provide slow-twitch, dystrophy-resistant tissue.

## Methods

### Cell culture and differentiation

Inducible iPax3 and iPax7 mouse embryonic stem (ES) cell lines were generated as previously described [16]. mES cells were maintained in knock-out DMEM (Invitrogen) supplemented with 15% FBS (Embryomax ES-qualified FBS—Millipore), 1% penicillin/streptomycin (Invitrogen), 2 mM glutamax (Invitrogen), 0.1 mM non-essential aminoacids (Invitrogen), 0.1 mM β-mercaptoethanol (Invitrogen), and 1000 U/ml LIF (Millipore). Skeletal myogenic differentiation was achieved as described [17]. Briefly, cells were detached and the supernatant was then incubated in an orbital shaker at 80 rpm at the concentration of 40000 cells/ml in embryoid body (EB) differentiation medium, composed of IMDM (Invitrogen) supplemented with 15% FBS (Embryomax ES-qualified FBS), 1% penicillin/streptomycin (Invitrogen), 2 mM glutamax (Invitrogen), 50 μg/ml ascorbic acid (Sigma-Aldrich), and 4.5 mM monothioglycerol (MP Biomedicals). Pax3 or Pax7 induction is induced starting from day 3 by administering 1 μM doxycycline (dox). Day 5 EBs were disaggregated, incubated with Fc block (1 μl/million cells, BD Biosciences) for 5 min and then with Flk1-APC and Vcam1-biotin-conjugated antibodies (1 μl/million cells, e-Bioscience) for 20 min on ice followed by 5-min incubation with streptavidin-PeCy7. Cells were washed twice with PBS and then resuspended in staining buffer (SB) composed of PBS containing 10% FBS and propidium iodide (PI) to exclude dead cells. Vcam1+FLK1- cells were sorted using a FACSAria II (BD Biosciences), replated on gelatin-coated dishes, and further expanded for 2–3 passages before collection for RNA extraction or transduced with H_2_B-GFP-encoding lentiviral vector for transplantation studies into NSG mice. For terminal differentiation, 50,000 cells were deposited onto a well of 24-well plates, allowed to reach confluence, and then cultured in EB media without dox for 5-7 days, after which cells were collected for RNA extraction or immunofluorescence staining.

### Labeling of myogenic progenitors

H_2_B-GFP-encoding lentiviral vectors were prepared as described [9]. Briefly, vectors were co-transfected with packaging plasmids Δ8.91 and pVSVG into 293T cells using the LTX transfection reagent (Thermofisher Scientific). Lentiviral-containing supernatant was collected 48 h after transfection, filtered, and used for transduction of myogenic progenitors upon centrifugation for 1.5 h at 2500 rpm and 37 °C.

### Primary cell isolation and transplantation studies

Animal experiments were carried out in strict accordance to protocols approved by the University of Minnesota Institutional Animal Care and Use Committee. Primary embryonic and fetal myoblasts were isolated and dissected from E10.5 and E14.5 Myf5Cre-Rosa26YFP [5, 6] mouse embryos and fetuses, respectively, while neonatal and adult satellite cells were isolated from hindlimbs of Pax7-ZsGreen mice [7] as previously described [10]. For RNA extraction and transplantation studies, we used YFP+ cells for prenatal myoblasts, ZsGreen+ cells for postnatal satellite cells, and CD31-/CD45-/Itga7+/Vcam1+/GFP+ for PSC donor-derived satellite cells. Freshly isolated ZsGreen+ cells were also seeded on gelatin-coated multiwell plates in the presence of EB medium and cultured for 5 to 7 days to induce terminal differentiation, which was followed by immunofluorescence analyses.

For transplantation studies, hindlimbs of 6–8-week-old male NOD-scid IL2Rgnull (NSG) and NSG^*mdx4cv*^ [14] mice were irradiated with a 12-Gy single dose at 24 h prior to injury of both tibialis anterior (TA) muscles with 15 μl of cardiotoxin 10 μM (Latoxan). One day later, iPax myogenic progenitors and freshly isolated embryonic and fetal myoblasts were resuspended in PBS at the concentration of 3 × 10^4^ cells/μl, while freshly isolated neonatal and satellite cells were resuspended between 3 and 5 × 10^2^ cells/μl. Donor-derived iPax mononuclear cells (iPax MNCs) were re-isolated 4 weeks after the transplantation of PSC-derived myogenic progenitors into primary recipients, as previously described [10]. For secondary transplantation, iPax MNCs were resuspended in PBS at the concentration of 1.5 × 10^3^ cells/μl. Ten microliters of a given cell suspension were injected in each TA while the contralateral TA received the same volume of PBS as internal control. Four to 6 weeks after transplantation, mice were euthanized, and TAs collected for immunostaining analysis as previously described [15]. Briefly, muscles were frozen in isopentane cooled in liquid nitrogen, and serial 10-μm-thick cryosections were collected and analyzed.

### Immunofluorescence

Immunofluorescence staining was performed on fixed cultured cells and on unfixed TA cryosections as described [18]. Briefly, unfixed TA cryosections were permeabilized with 0.3% Triton/PBS, followed by blocking with 3% BSA/PBS before incubating with the primary antibodies for dystrophin (rabbit polyclonal 1:250, Abcam), MyHC type I (BA-D5), type IIA (sc-71), type IIB (BF-F3), type I+IIA+IIB (BF-35), all mouse monoclonal from DSHB, 1:100, ON at 4 °C. The following day, samples were rinsed with PBS and then incubated with the secondary antibodies goat anti-rabbit Alexa Fluor-488 or 647, and with goat anti-mouse Alexa Fluor-555 and DAPI for 1 h at RT. After washing three times with PBS, sections were briefly dried and mounted using Prolong Gold with DAPI (Invitrogen). Pictures were acquired with Axioimager M1 fluorescence microscope (Zeiss) and analyzed with ZEN Blue software. Quantification of positive myofibers and calculation of cross-sectional area (CSA) were performed using Fiji ImageJ software.

### RNA isolation and gene expression analyses

Samples for RNA were resuspended in Qiazol (Qiagen) and RNA extracted with the Qiagen MiRNeasy Mini Kit, according to manufacturer’s instructions. After RNA extraction, in column DNase digestion was performed and samples were retro-transcribed using Superscript Vilo (Invitrogen). Gene expression analyses were performed using an amount of cDNA corresponding to 5 ng of starting RNA for each reaction. RT-qPCR was performed using Premix Ex Taq (Probe qPCR) Master Mix (Takara) and TaqMan probes (Applied Biosystems).

### RNA sequencing and data availability

Sequencing data and corresponding analyses have been conducted previously [10]. Raw and processed data have been deposited to the NCBI Gene Expression Omnibus (GEO) database and are accessible under the GEO accession number GSE121639.

### Functional annotation

Gene lists were submitted to Gene Ontology Consortium (geneontology.org) and annotated for complete cellular components (GO CC) analyses. Top enriched categories were selected based on *p* value corrected for multiple hypothesis testing (B+H FDR). Logarithm of *p* value was then plotted using Microsoft Office Excel or Prism (Graphpad).

### Statistical analysis

Differences between samples were assessed by using the unpaired two-tailed Student’s *t* test for single comparisons, or one-way ANOVA followed by post hoc Tukey test among multiple groups. *p* values < 0.05 were considered statistically significant.

## Results

### Predominance of slow and oxidative fibers in primary transplants by PSC-derived myogenic progenitors

**Fig. 1:**
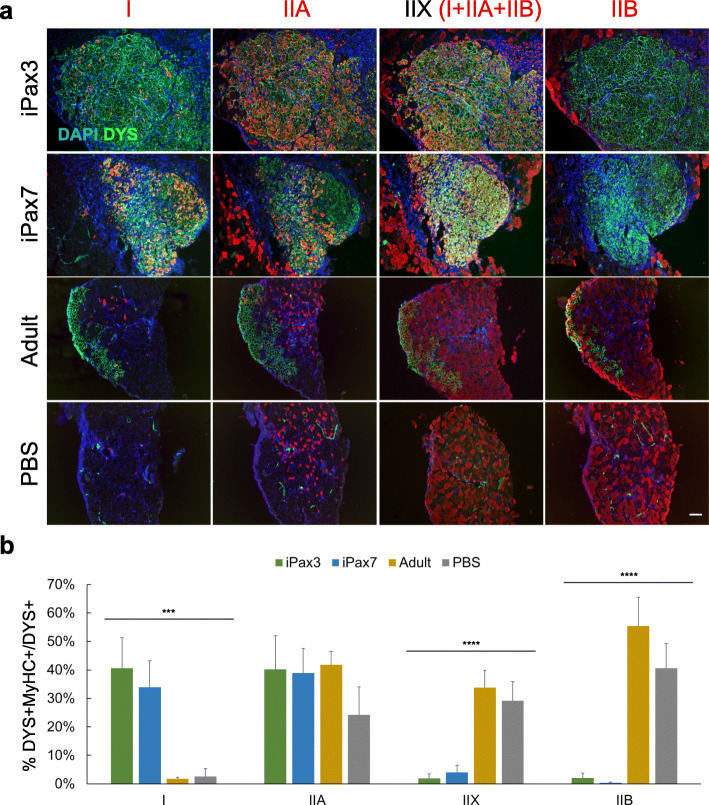
PSC-derived myogenic progenitors preferentially give rise to myofibers expressing oxidative MyHC isoforms. **a** Representative images show staining for MyHC isoforms (red) and dystrophin (green) in NSG*mdx*^*4cv*^. Nuclei were counterstained with DAPI (blue). Magnification bar, 100 μm. **b** Bar graphs show the percentage of DYS+MyHC+ double positive with respect to total donor-derived DYS+ myofibers. Data are shown as mean ± SEM (*n* = 7–8 per group). ***p < 0.001 and ****p < 0.0001. Adult, satellite cells from TAs of 3-month-old NSG*mdx*^*4cv*^ mice

To assess the in vivo fiber type composition produced by PSC-derived myogenic progenitors, we generated myogenic progenitors from dox-inducible (i)Pax3 and iPax7 mouse embryonic stem cells [8, 9]. These myogenic progenitors were then transplanted into pre-injured tibialis anterior (TA) muscles of immunocompromised dystrophic NSG*mdx*^*4cv*^ mice [19]. As reference control, we performed a similar set of transplantations using satellite cells freshly isolated from TAs of 3-month-old Pax7-ZsGreen mice [20]. Our immunofluorescence results show that both iPax3 and iPax7 myogenic progenitors give rise to a high proportion of slow-twitch type I, as indicated by the expression of MyHC type I when compared to muscles injected with adult satellite cells or PBS alone (Fig. 1a, b). Similar results were observed using the in vitro differentiation system, demonstrating that the type I MyHC isoform is expressed in PSC-derived myotubes, unlike samples derived from adult satellite cells freshly isolated from TAs (Fig. S1). As shown in Fig. 1a and b, fast-twitch glycolytic fibers IIX and IIB were detected at a much lower frequency in iPax3- and iPax7-injected muscles. Consequently, the ratio of total oxidative (I+IIA) myofibers over the total glycolytic (IIX+IIB) myofibers is higher in PSC-derived myofibers (Fig. S2a). Of note, engraftment from adult satellite cells exhibits the same fiber type composition observed in PBS-injected controls, which is what is expected in TA muscles, mainly composed of type II fast-twitch fibers [21]. We observed the same phenotype (Fig. S2b), when NSG*mdx*^*4cv*^ mice were injected with satellite cells isolated from the slow-twitch *soleus* muscle [3], corroborating published data suggesting that satellite cells do not retain a heritable fiber type phenotype [22]. These data suggest that, unlike primary satellite cells, iPax3 and iPax7 PSC-derived myogenic progenitors preferentially express type I MyHC and that they do not acquire the fiber type composition of recipient muscles.

Due to the embryonic nature of PSC-derived myogenic progenitors [10], a high number of iPax3- and iPax7-derived myofibers express embryonic MyHC (Fig. S2c-d), in contrast to adult satellite cell-derived myofibers. Consistently, cross-sectional area (CSA) analysis shows that myofibers from PSC-derived myogenic progenitor transplants display smaller size than those produced by adult satellite cells, as a high frequency of myofibers is smaller than 100 μm^2^ (Fig. S2e).

Since in vitro-generated iPax3 and iPax7 PSC-derived myogenic progenitors display a prenatal molecular signature profile [10], we also determined myofiber type engraftment of primary cells isolated from E10.5 embryos (embryonic), E14.5 fetuses (fetal), and 3-day-old pups (neonatal) samples (Fig. S3a). Embryonic myoblasts give rise to a significantly lower proportion of type IIB myofibers compared to fetal and neonatal satellite cells, in agreement with published data [23]. Nevertheless, they do not show a preferential expression of slow and oxidative MyHC isoforms, as observed with myofibers generated by iPax3/iPax7 myogenic progenitors, thus suggesting that this is a unique feature of PSC-derived myofibers rather than an earlier developmental characteristic.

Altogether, these data suggest that PSC-derived progenitors preferentially give rise to a high proportion of slow-twitch type I and fast-twitch type IIA oxidative myofibers.

### Fiber type composition of PSC-derived skeletal muscle is cell-autonomous in primary transplants

**Fig. 2:**
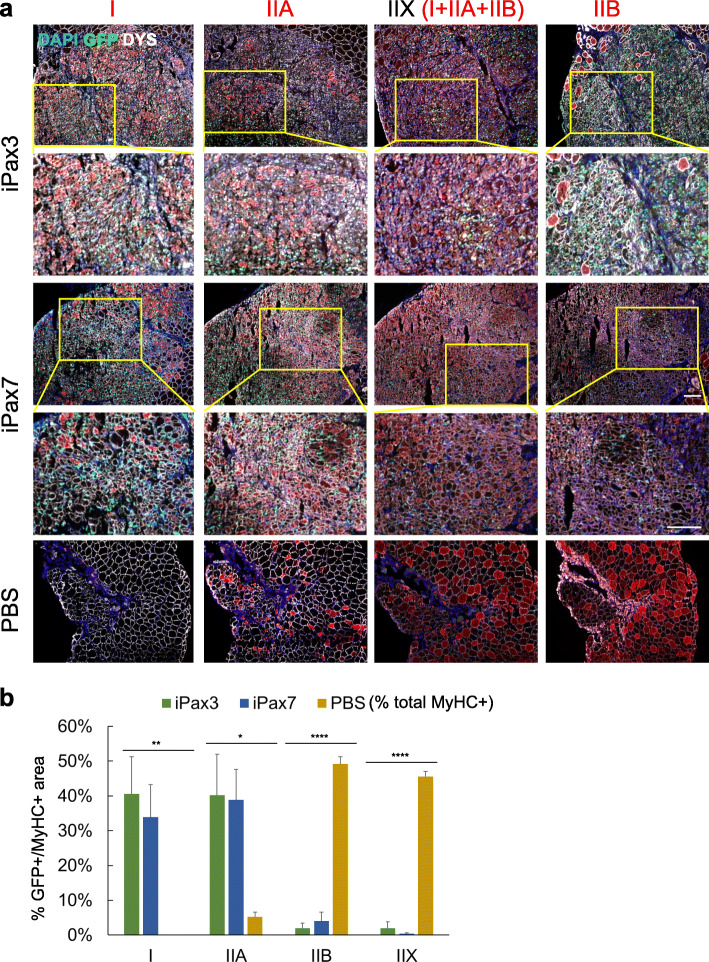
Fiber type composition of PSC-derived skeletal muscle is not dependent on the background of recipient muscles. **a** Representative images show staining for MyHC isoforms (red) and dystrophin (white) in TA muscles from NSG mice that had been injected with iPax3 or iPax7 myogenic progenitors. PBS served as control. Green nuclei represent epifluorescence of H_2_B-GFP. Nuclei were counterstained with DAPI (blue). Yellow boxes indicate position of the higher magnification insets below. Magnification bar, 100 μm. **b** Bar graphs show respective quantification (panel **a**). Percentages represent the ratio between the engraftment area (GFP) and MyHC expression, in comparison to PBS, represented as the percentage of total MyHC positive. Data are shown as mean ± SEM (*n* = 4 per group). **p < 0.05, ***p < 0.001, and ****p < 0.0001

It has been suggested that dystrophic muscles show a higher proportion of oxidative, type I myofibers with respect to wild-type (WT) controls, and this phenomenon has been ascribed to a slower degeneration rate of the slow-twitch fibers in pathologies such as DMD and FSHD [7, 14, 15]. In agreement, we observed a similar phenotype in TA muscles of NSG*mdx*^*4cv*^ mice, which show type I MyHC-positive myofibers, unlike age- and gender-matched muscles from NSG mice (Fig. S4a-b). Of note, the relative proportion of all analyzed myofibers is significantly different between the two mouse strains, with dystrophic mice showing more oxidative myofibers than NSG mice, while the number of glycolytic myofibers is significantly lower, as shown in Fig. S4b. Based on these observations, we sought to determine whether the observed high proportion of PSC-derived type I myofibers is due to the dystrophic environment. To test this hypothesis, we performed transplantation studies in non-dystrophic recipients. As shown in Fig. 2a, PSC-derived myogenic progenitors injected in a non-dystrophic environment generated preferentially slow and oxidative myofibers. Quantification of the engrafted tissue, as indicated by PSC-derived GFP expression with respect to the MyHC expression area (Fig. 2b), revealed a trend of fiber type composition similar to the one observed in dystrophic muscles (Fig. S4c). Of note, transplantation of PSC-derived myofibers into non-dystrophic mice resulted in a higher frequency of type IIA MyHC fibers with respect to the host environment (Fig. 2b), and this is probably due to the low numbers of IIA myofibers found in NSG TA muscles.

Altogether, these data suggest that iPax3/iPax7 PSC-derived skeletal myogenic progenitors give rise to specific myofiber types independently from the host environment.

### PSC-derived myofibers express genes associated with mitochondrial function

**Fig. 3:**
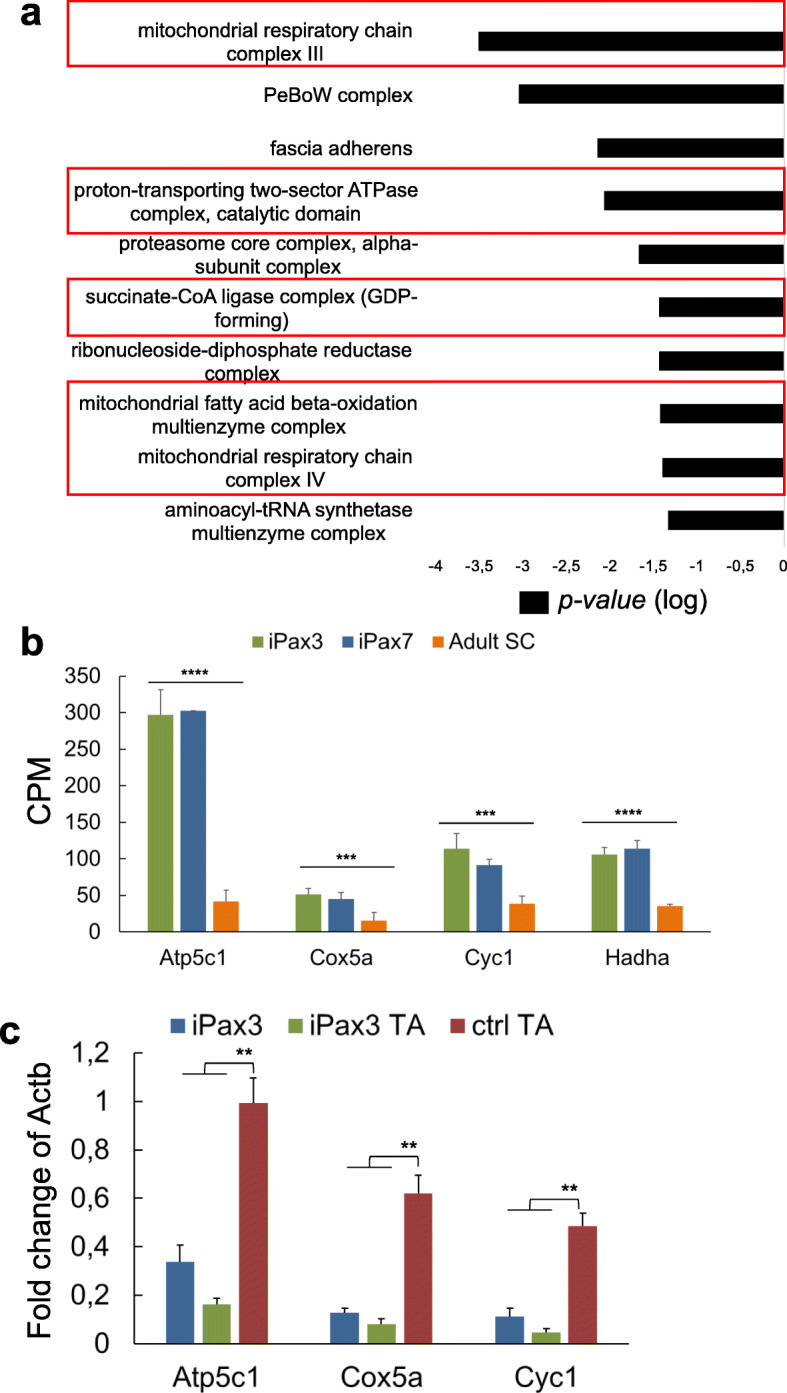
PSC-derived skeletal muscle fibers express genes associated with mitochondrial function. **a** Plot shows gene ontology cellular components terms, ranked based on hierarchy, of the genes upregulated in PSC-derived iPax3 and iPax7 myogenic progenitors. Black bars indicate *p* value in logarithmic scale representing B+H FDR. Red boxes highlight mitochondrial specific categories. **b** Graphs show the average count per million (CPM) values for representative differentially expressed genes from the categories highlighted in panel **a**. Data are shown as mean ± SEM (*n* = 4 per group). ***p < 0.001 and ****p < 0.0001. **c** Graph shows RT-qPCR results of mRNA expression levels of the genes for ATP synthase, H+ transporting, mitochondrial F1 complex, gamma polypeptide 1 (*Atp5c1*), cytochrome c oxidase subunit 5A (*Cox5a*), and cytochrome c1 (*Cyc1*). Data are shown as mean ± SEM (*n* = 4 per group). **p < 0.01. iPax3 TA, NSG*mdx*^*4cv*^ muscles injected with iPax3 myogenic progenitors; ctrl TA, non-injected muscles of 3-month-old NSG*mdx*^*4cv*^ mice

As slow and oxidative fibers are characterized by oxidative metabolism [3], we reasoned that genes related to mitochondria function would be highly expressed in PSC-derived myogenic progenitors and myofibers. Before assessing the in vivo gene expression of engrafted PSC-derived myofibers, we took advantage of our transcriptome study on in vitro*-*generated cells [10] to investigate whether differences in the expression of metabolic genes are already visible at the myogenic progenitor stage. Gene ontology analysis of the genes upregulated in iPax3/7 myogenic progenitors with respect to adult satellite cells demonstrated that mitochondrial complexes represent half of the top ten categories based on cellular component classification (red boxes, Fig. 3a). Accordingly, genes encoding for known components of the mitochondrial respiratory machinery, such as the proton-transporting two-sector ATPase complex (*Atp5c1*), mitochondrial chain complex III (*Cyc1*), and IV (*Cox5a*), and the mitochondrial fatty acid beta-oxidation multienzyme complex (*Hadha*), are highly expressed in iPax3 and iPax7 PSC-derived myogenic progenitors when compared to adult satellite cells (SC, Fig. 3b). To verify whether this transcriptional profile is maintained in PSC-derived myofibers, we performed gene expression analyses in engrafted TA muscles that had been injected with iPax3 myogenic progenitors. As shown in Fig. 3c, the expression levels of genes associated with mitochondrial function, including *Atp5c1*, *Cox5a,* and *Cyc1,* are similar between PSC-derived in vitro-differentiated myotubes (iPax3) and in vivo-generated myofibers (iPax3 TA). However, the expression levels of these genes are significantly lower when compared to control non-injected TA muscles from NSG*mdx*^*4cv*^ (ctrl TA).

These data suggest that in vitro-generated myogenic progenitors are endowed with oxidative properties, which are maintained upon in vivo differentiation into myofibers.

### Exposure to the muscle environment does not influence the oxidative phenotype of PSC-derived myofibers in secondary transplants

**Fig. 4:**
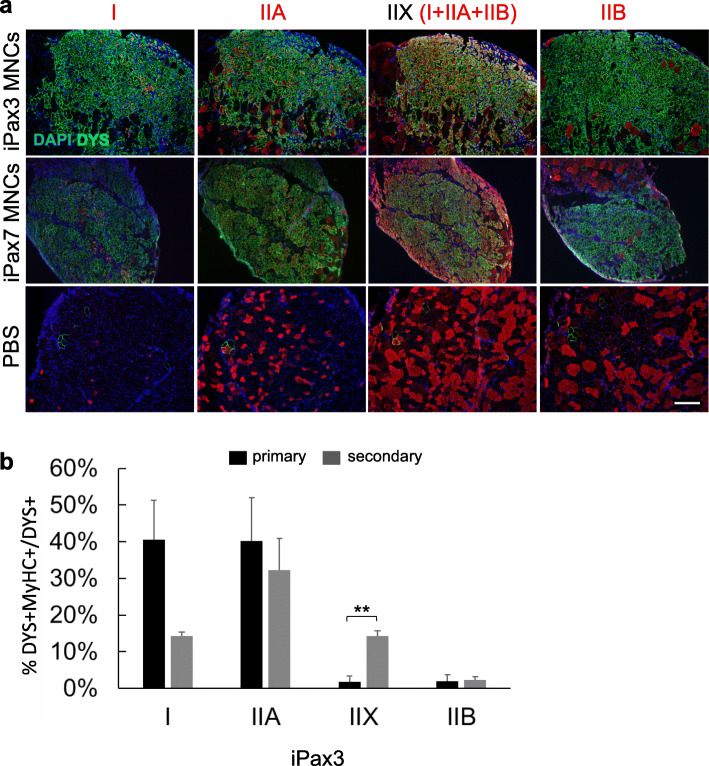
Exposure to the adult endogenous environment does not influence the oxidative phenotype of PSC-derived myofibers. **a** Representative images show staining for MyHC isoforms (red) and dystrophin (green) in TA muscles from NSG*mdx*^*4cv*^ mice that had been injected with iPax3 or iPax7-derived re-isolated mononuclear cells (MNCs). PBS served as control. Nuclei were counterstained with DAPI (blue). Magnification bar, 100 μm. **b** Bar graphs show respective quantification (panel **a**), as indicated by the percentage of DYS+MyHC+ double positive with respect to total DYS+ myofibers for iPax3-injected MNCs (gray bar, secondary recipients) in comparison to data from iPax3-injected myogenic progenitors (black bars, primary recipients). Data are shown as mean ± SEM (*n* = 4 per group). **p < 0.01

Along with developmental MyHCs, type I MyHC is abundant in primary prenatal myofibers [24]. Since PSC-derived myogenic progenitors show a prenatal molecular signature, and exposure to the endogenous muscle environment induce their maturation towards a postnatal phenotype [10], we assessed whether the high expression of MyHC type I in PSC-derived myofibers is due to the prenatal developmental stage of these cells. To test this, we re-isolated iPax3/iPax7 donor-derived MNCs from primary recipient muscles and injected these into secondary dystrophic recipients. Our results show that both type IIA and IIB MyHC isoforms are expressed at similar levels in primary and secondary engrafted PSC-derived myofibers. The frequency of type I isoform is lower in secondary recipients, but this is not significantly different from primary grafts (*p* = 0.064, Fig. 4a). Of note, we observed reduced frequency of embryonic MyHC-positive fibers (Fig. S5a-b). These data suggest that the preferred composition of oxidative myofibers is not due to the immature phenotype of the primary grafts but rather due to an intrinsic property of iPax3 and iPax7-derived skeletal muscle. Importantly, muscles injected with re-isolated MNCs display higher frequency of glycolytic fast type IIX myofibers compared to their primary engrafted counterparts (Fig. 4). Type IIX MyHC is only expressed in myofibers after birth, arising from the secondary myogenesis wave [24]. Therefore, the maturation of PSC-derived myogenic progenitors, occurring upon exposure to the endogenous muscle environment, is also reflected in the generation of postnatal myofibers.

Altogether, these results demonstrate that PSC-derived myogenic progenitors have an intrinsic capacity to preferentially give rise to oxidative myofibers.

## Discussion

Several studies have suggested that disease pathogenesis due to neuromuscular disorders can affect fiber type composition and therefore muscle properties, such as endurance and resistance to fatigue [6, 25]. Within this heterogeneous group of disorders, DMD is characterized by highly fibrotic skeletal muscle [26] and increased number of slow myofibers, which display higher resistance to pathogenesis, while glycolytic fast fibers progressively vanish with disease progression [7]. Therefore, when developing a cell replacement strategy for skeletal muscle disorders, it is important to determine the fiber type composition of engrafted cells.

In this study, we investigated the content of myofiber types generated following the transplantation of PSC-derived myogenic progenitors. We focused on mouse-to-mouse engraftment to prevent species-specific differences that could introduce bias to our data, as human muscles lack the type IIB MyHC isoform [4]. Our results showed that PSC-derived engrafted muscle preferentially expresses types I and IIA MyHC isoforms, which are features of slow and/or oxidative myofibers [3]. Our data also show that a high number of PSC-derived myofibers express the embryonic MyHC isoform, indicating that the prenatal molecular signature of iPax3 and iPax7 PSC-derived myogenic progenitors is likely maintained in their respective regenerating muscle in primary grafts [10]. Based on this observation, we hypothesized that the high proportion of oxidative myofibers could be ascribed to two different reasons: (1) PSC-derived myofibers generate oxidative fibers as an indirect result of the pressure from the dystrophic environment, which delays degeneration of diseased slow-twitch fibers, (2) PSC-derived myofibers are immature because they derive from myogenic progenitors with a prenatal molecular signature, hence they express more type I and IIA MyHC isoforms, which arise earlier during developmental myogenesis [24].

To rule out the influence of the recipient environment, we transplanted non-dystrophic mice and compared the pattern of fiber type composition to engrafted muscles from dystrophic mice. Our data show that PSC-derived myofibers express more type I MyHC, regardless of the recipient genetic background, suggesting this is a cell-autonomous feature of the donor cells. Accordingly, we interrogated the transcriptome of PSC-derived iPax3 and iPax7 myogenic progenitors [10], and we found that mitochondrial complexes are the most represented cellular components in both cell lines, which is in accordance with oxidative metabolic needs. Moreover, we found that representative genes encoding for proteins of mitochondrial complexes are also expressed at similar levels in PSC-derived in vitro myotubes and in vivo myofibers, suggesting that PSC-derived myogenic progenitors maintain oxidative properties upon in vivo differentiation to myofibers. However, we also observed that non-injected dystrophic muscles express higher levels of the selected mitochondrial genes. These data indicate that the myofibers generated from PSC-derived myogenic progenitor, although expressing genes related to mitochondrial activity, may have a metabolic profile proper of immature organelles, as recently shown for fetal and neonatal satellite cells [27]. In the same paper, Pala et al. suggest that mouse myogenic progenitors at different developmental and functional stages have different metabolic requirements, and metabolic reprogramming may influence the progression throughout these stages. Of note, human PSC-derived myogenic progenitors have been described to display fetal characteristics [18, 28, 29]. Therefore, future studies should focus on fiber type composition and metabolic properties of human PSC-derived myogenic progenitors.

To further assess whether the level of maturation of transplanted cells impacts the fiber type composition of engrafted skeletal muscle, we re-isolated the donor-derived MNC fraction from primary grafts and transplanted these into secondary recipient mice. Our results show that engrafted muscle in secondary recipients displays an increased proportion of glycolytic fast-twitch fiber IIX with respect to primary counterparts. Of note, they also show lower number of fibers expressing the embryonic MyHC isoform. These results reinforce and complement our recent data demonstrating that the maturation of iPax3 and iPax7 myogenic progenitors occurs upon exposure to the endogenous adult muscle environment [10]. However, when looking at the numbers of slow and oxidative donor-derived myofibers, we found that these are comparable between primary and secondary engrafted muscle. This observation suggests that the prenatal molecular signature of in vitro-generated PSC-derived myogenic progenitors might be only marginally responsible for the high number of slow-twitch and oxidative myofibers found upon engraftment, although we cannot exclude that the donor-derived MNC fraction did not sufficiently mature after the 4-week-exposure to the endogenous adult muscle environment.

When generating cells suitable for replacement therapies in the context of skeletal muscle, several characteristics should be evaluated, including engraftment and regenerative capacity, ability to repopulate the satellite cell compartment, and the long-term contribution to the recipient tissue’s functionality. One of the key functions of skeletal muscle is the ability to respond to innervation stimuli and to contract according to specific endurance and speed abilities, which are unique to different groups of muscles, but highly plastic. To achieve this level of complexity, it is fundamental to assess the ability of in vitro-generated myogenic progenitors to contribute to the tissue physiology, thus ensuring proper integration into the host environment. Up to date, several studies have assessed the therapeutic properties of muscle cells from different sources [30]. However, very few studies have analyzed the fiber type specification of cells generated in vitro [11, 12, 31], and only one assessed the contribution of transplanted cells to fiber type specification in vivo [13], which showed that myofibers generated by PSC-derived teratomas preferentially express type I, IIA, and IIB MyHC isoforms, while IIX myofibers were not detected.

The present study documents for the first time the in vivo myofiber composition resulting from the transplantation of in vitro-generated PSC-derived myogenic progenitors. Our findings show that donor-derived myofiber contribution by PSC-derived myogenic progenitors is characterized by the expression of slow and oxidative myosin heavy-chain isoforms, in addition to developmental myosins, and that transplantation of the donor-derived MNC fraction into secondary recipients results in myofibers still predominantly expressing slow and oxidative myosin heavy-chain isoforms, whereas expression of developmental myosins is reduced and type IIX is increased. Taken together, our data suggest that exposure to the adult muscle environment favors postnatal myofiber composition switch and that elevated oxidative fiber type composition is a cell-autonomous characteristic of iPax3 and iPax7 PSC-derived myofibers, which could be relevant for the development of cell replacement therapy for DMD.

## Conclusions

We have shown that iPax3 and iPax7 PSC-derived myogenic progenitors cell-autonomously generate slow and oxidative myofibers upon transplantation into dystrophic mice. This capacity is maintained upon re-isolation of donor-derived MNCs and their injection into secondary recipients. If these findings are recapitulated with human PSC-derived myofibers, these results would suggest that in vitro-generated myogenic progenitors could have a double benefit in the therapy of DMD: they may (i) replace diseased skeletal muscle and (ii) provide myofibers more resistant to disease progression.

## Supplementary information

**Additional file 1: Figure S1.** Fiber type composition of myotubes resulting from the *in vitro* differentiation of PSC-derived iPax3/iPax7 myogenic progenitors and adult satellite cells. Representative images show immunofluorescence staining for pan, embryonic, type I and IIA MyHC isoforms (red) in myotubes resulting from the *in vitro* differentiation of iPax3 and iPax7 PSC-derived myogenic progenitors, as well as adult satellite cells (adult). Nuclei were counterstained with DAPI (blue). Magnification bar: 100 μm. **Figure S2.** Characterization of fiber type composition. a) Graph shows the ratio of oxidative DYS+MyHC+ type I and type IIA over glycolytic type IIX and IIB myofibers following the transplantation with iPax3 and iPax7 myogenic progenitors, and adult satellite cells. Data are shown as mean ± SEM (n = 7-8 per group). **p < 0.01. b) Graph bars show percentage of DYS+MyHC+ double positive with respect to total DYS+ in TA muscles transplanted with satellite cells isolated from TA or soleus muscles. Data are shown as mean ± SEM (n = 4 per group) and no statistically significant differences were observed among groups. c) Representative images show staining for embryonic MyHC (red) and dystrophin (green) in TA muscles from NSG*mdx*^*4cv*^ mice that had been injected with iPax3 or iPax7 myogenic progenitors. Adult satellite cells served as control. Magnification bar: 100 μm. d) Graph shows respective quantification (panel c), as indicated by the number of DYS+MyHC+ double positive with respect to total DYS+. Data are shown as mean ± SEM (n = 6-8 per group). *p < 0.05 and ***p < 0.001. d) Quantification of CSA (μm^2^) of grafts from indicated groups. Data are shown as mean ± SEM (n = 4-5 per group). *p < 0.05. **Figure S3.** Characterization of fiber type composition following the transplantation of embryonic, fetal, and neonatal primary cells. a) Representative images show staining for MyHC isoforms (red) and dystrophin (green). Nuclei were counterstained with DAPI (blue). Magnification bar: 100 μm. b) Percentage quantification of DYS+MyHC+ double positive with respect to total donor-derived DYS+ myofibers. Data are shown as mean ± SEM (n = 4-5 per group) **p < 0.01. **Figure S4.** Characterization of fiber type composition based on recipient genetic background. a) Representative images show staining for type I MyHC (red) and Laminin (green) in TA muscles from dystrophic NSG*mdx*^*4cv*^ and NSG mice. b) Graph shows respective quantification (panel a), as indicated by the number of MyHC+ myofibers in TA muscles from dystrophic NSG*mdx*^*4cv*^ and NSG mice. Data are shown as mean ± SEM (n = 6 per group). *p < 0.05, **p < 0.01, and ***p < 0.001. c) Graph bar shows percentage of MyHC+ donor-derived myofibers from Fig. 2a in comparison to iPax3 and iPax7 samples from Fig. 1b. Data are shown as mean ± SEM (n = 6-8 per group), and no statistically significant differences were observed among groups. Figure S5. Characterization of fiber types from secondary grafts. a) Representative image shows staining for embryonic MyHC (red) and dystrophin (green) in TA muscles of secondary recipients that had been injected with iPax3-derived MNCs. Magnification bar: 100 μm. b) Graph shows respective quantification (panel a), as indicated by the number of DYS+MyHC+ double positive with respect to total DYS+ myofibers of secondary grafts (iPax3 MNCs) in comparison to primary-injected TAs (iPax3). Data are shown as mean ± SEM (n = 4 per group). **p < 0.01.

## Data Availability

The datasets used during the current study are deposited and publicly available. Materials used in this study are commercially available.

## Abbreviations

CSA: Cross-sectional area
DAPI: 4,6-diamidino-2-phenylindole
DM1-2: Myotonic dystrophy 1 and 2
DMD: Duchenne muscular dystrophy
FSHD: Facioscapulohumeral muscular dystrophy
H2B-GFP: Histone 2B-green fluorescent protein
iPax3/7: Inducible Pax3/7
MyHC: Myosin heavy chain
MNC: Mononuclear cell
NSG: NOD-scid IL2Rgnull
PBS: Phosphate-buffer saline
PSC: Pluripotent stem cells
SB: Staining buffer
TA: Tibialis anterior
