# Supplementary figures and images for: Pluripotent stem cell-derived skeletal muscle fibers preferentially express myosin heavy-chain isoforms associated with slow and oxidative muscles
Source: Skelet Muscle. 2020 Jun 3;10:17. doi: 10.1186/s13395-020-00234-5 (PMC7268645; doi:10.1186/s13395-020-00234-5)

Figure S1

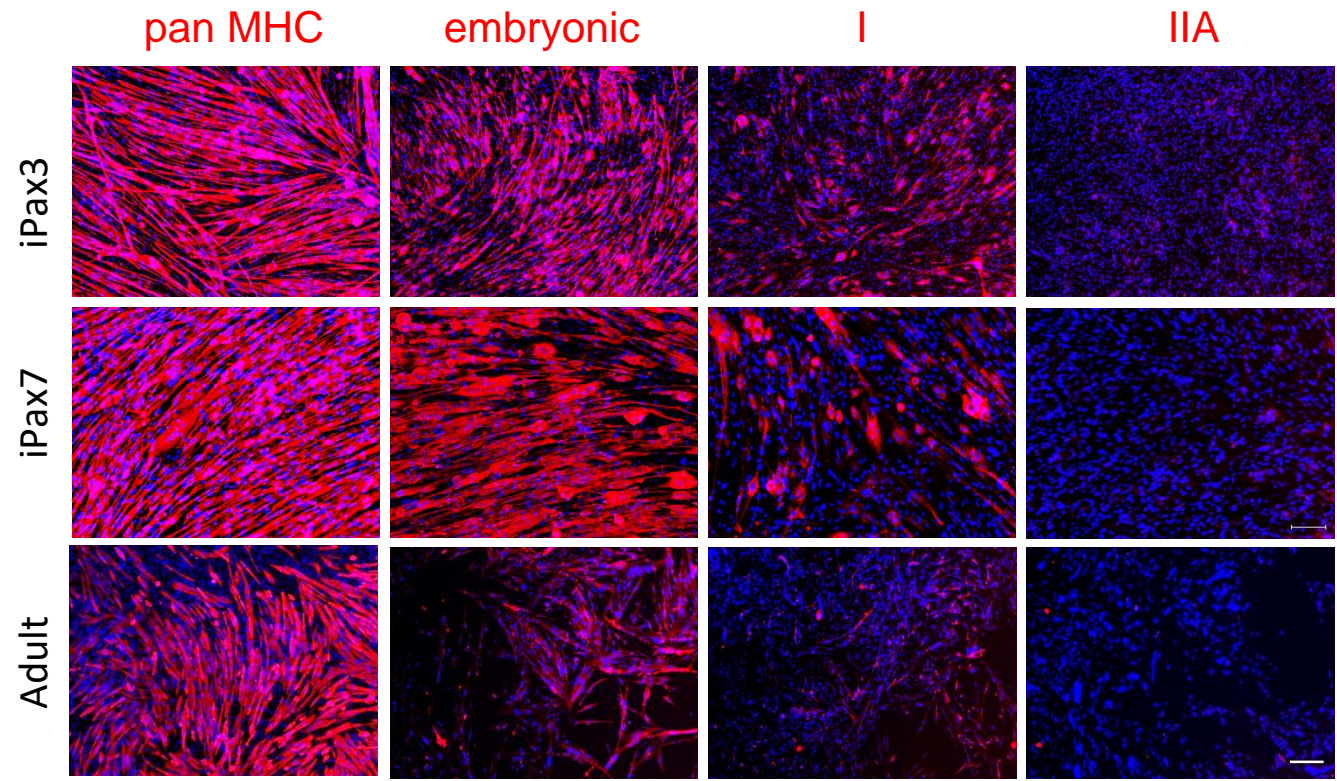

Figure S2

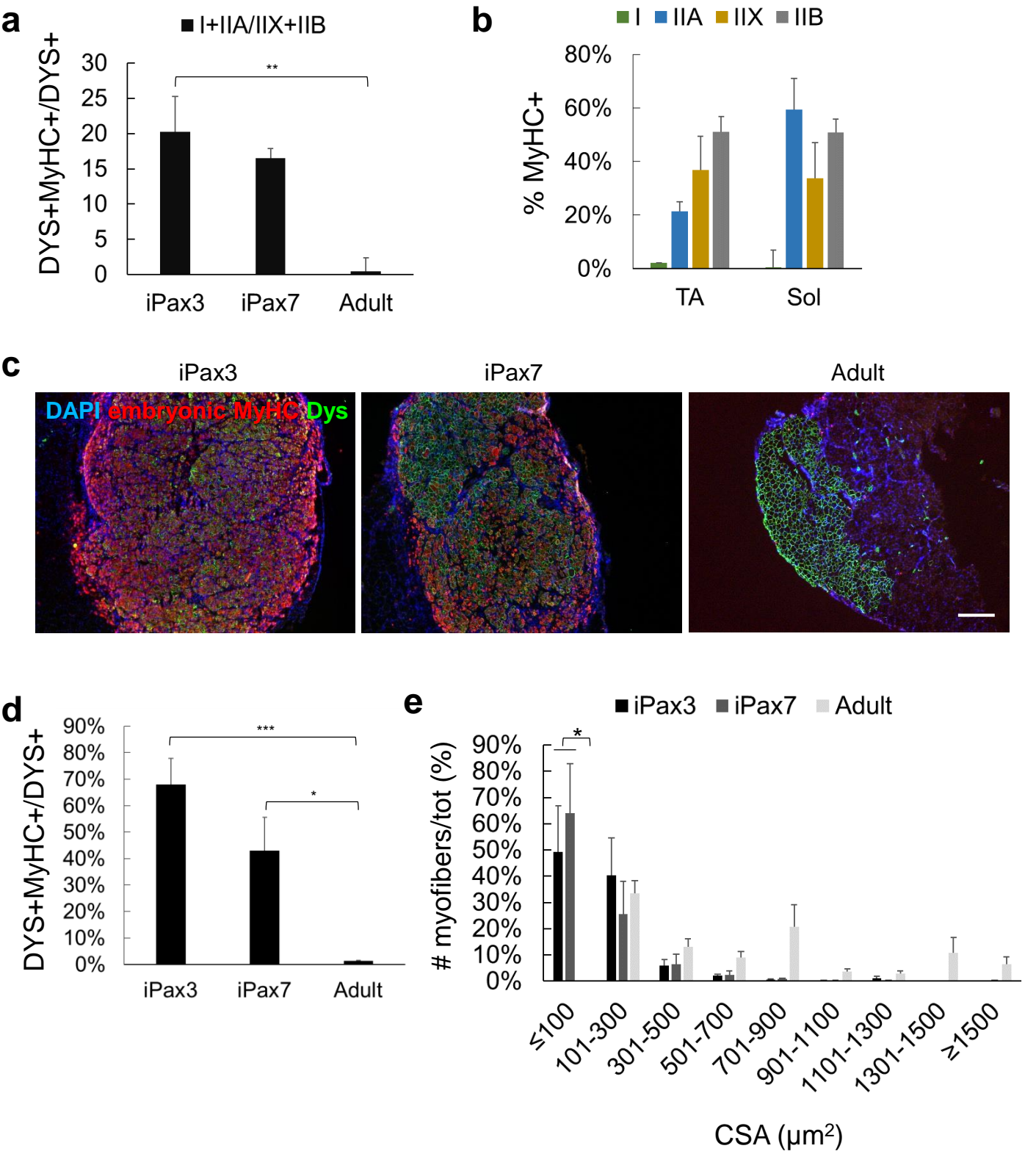

Figure S3

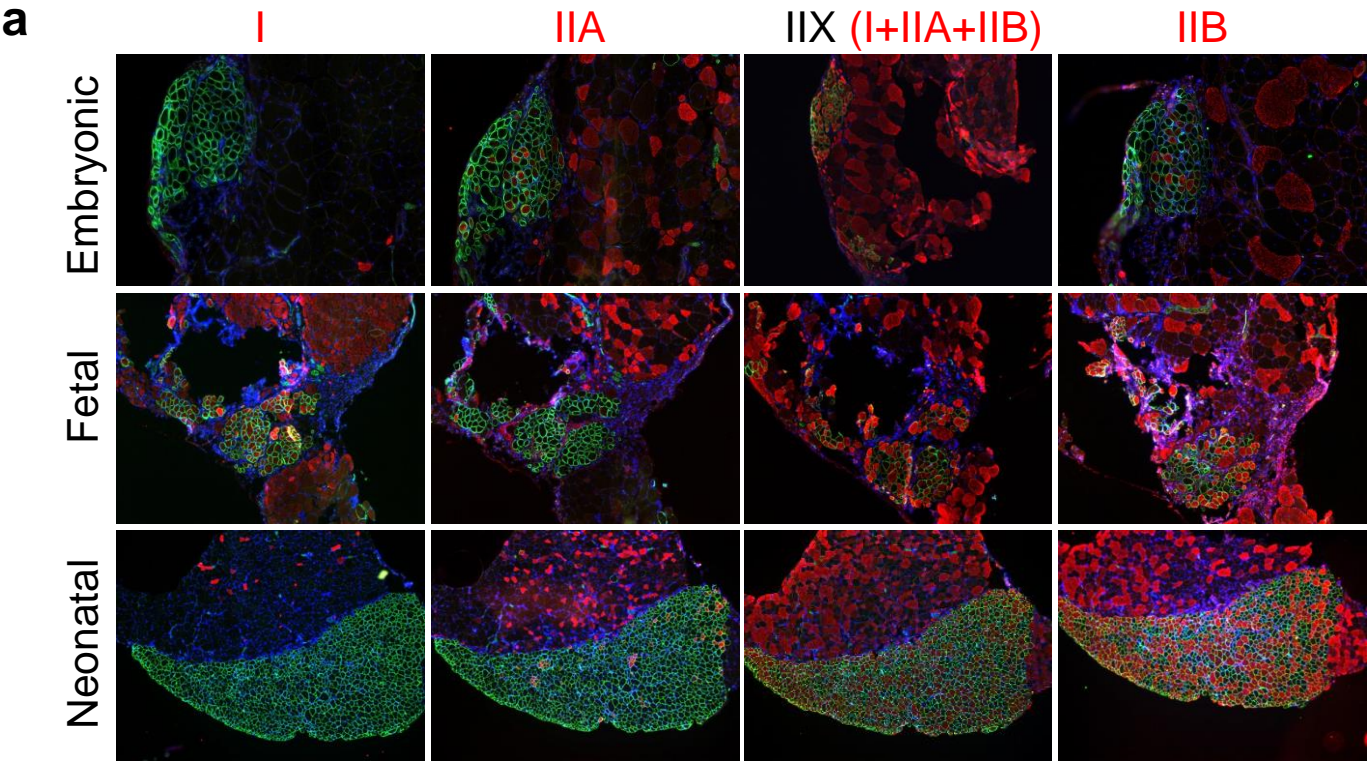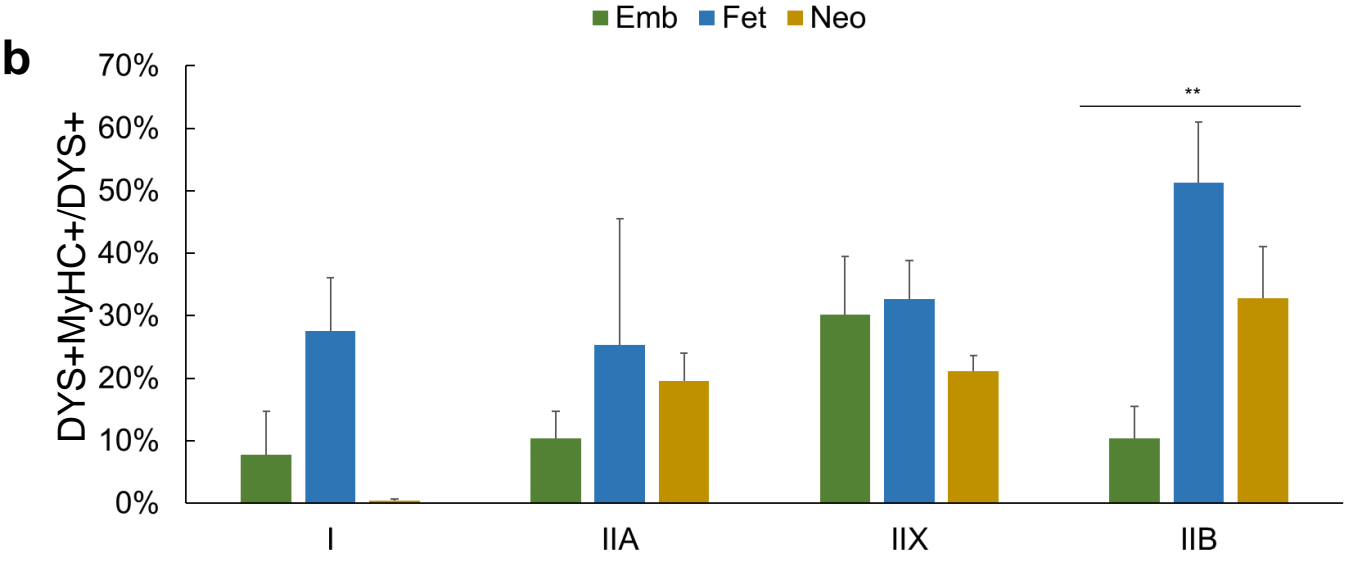

Figure S4

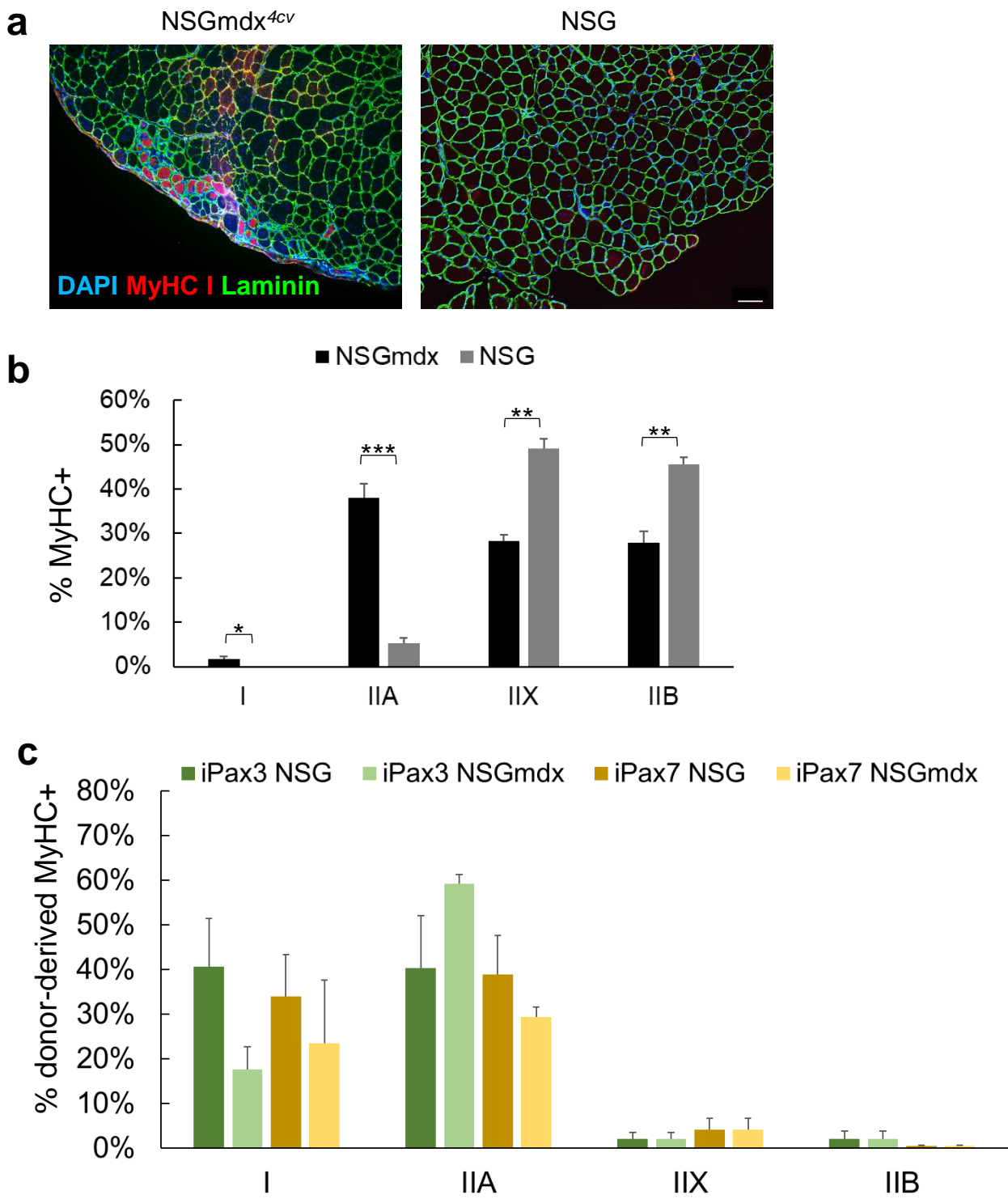

Figure S5

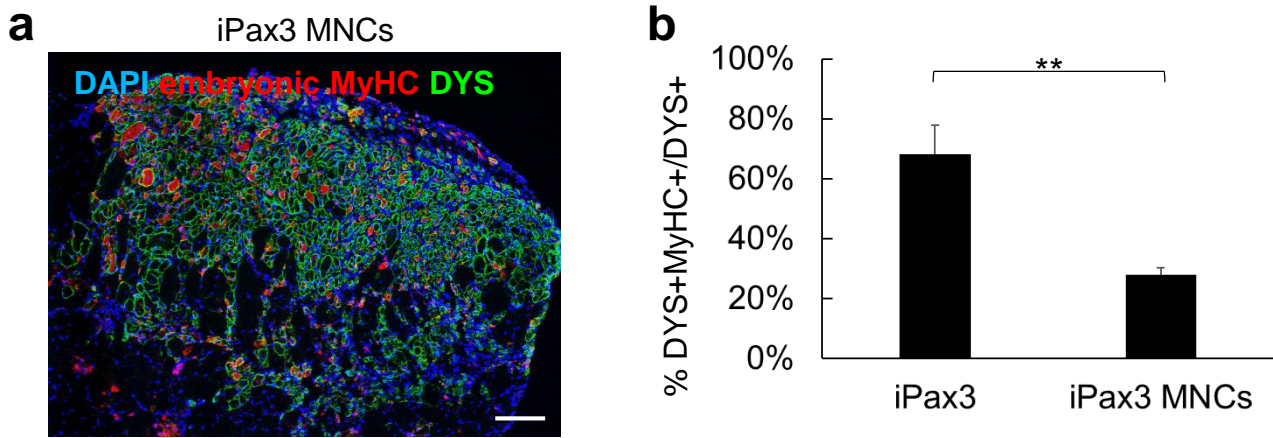

Supplement: Supplementary file 1 — Additional file 1: Figure S1. Fiber type composition of myotubes resulting from the in vitro differentiation of PSC-derived iPax3/iPax7 myogenic progenitors and adult satellite cells. Representative images show immunofluorescence staining for pan, embryonic, type I and IIA MyHC isoforms (red) in myotubes resulting from the in vitro differentiation of iPax3 and iPax7 PSC-derived myogenic progenitors, as well as adult satellite cells (adult). Nuclei were counterstained with DAPI (blue). Magnification bar: 100 μm. Figure S2. Characterization of fiber type composition. a) Graph shows the ratio of oxidative DYS+MyHC+ type I and type IIA over glycolytic type IIX and IIB myofibers following the transplantation with iPax3 and iPax7 myogenic progenitors, and adult satellite cells. Data are shown as mean ± SEM (n = 7-8 per group). **p < 0.01. b) Graph bars show percentage of DYS+MyHC+ double positive with respect to total DYS+ in TA muscles transplanted with satellite cells isolated from TA or soleus muscles. Data are shown as mean ± SEM (n = 4 per group) and no statistically significant differences were observed among groups. c) Representative images show staining for embryonic MyHC (red) and dystrophin (green) in TA muscles from NSGmdx4cv mice that had been injected with iPax3 or iPax7 myogenic progenitors. Adult satellite cells served as control. Magnification bar: 100 μm. d) Graph shows respective quantification (panel c), as indicated by the number of DYS+MyHC+ double positive with respect to total DYS+. Data are shown as mean ± SEM (n = 6-8 per group). *p < 0.05 and ***p < 0.001. d) Quantification of CSA (μm2) of grafts from indicated groups. Data are shown as mean ± SEM (n = 4-5 per group). *p < 0.05. Figure S3. Characterization of fiber type composition following the transplantation of embryonic, fetal, and neonatal primary cells. a) Representative images show staining for MyHC isoforms (red) and dystrophin (green). Nuclei were counterstained with DAPI (blue). Magnifi [file 13395_2020_234_MOESM1_ESM.pdf]
